# On the Cure of Erysipelas of the Face by the External Application of the Nitrate of Silver

**Published:** 1827-09

**Authors:** John Higginbottom

**Affiliations:** Member of the Royal College of Surgeons of London.


### ERYSIPELAS.

*On the Cure of Erysipelas of the Face by the external Application of the Nitrate of Silver.* By John Higginbottom, Esq. Member of the Royal College of Surgeons of London.

In my investigation of the use of the nitrate of silver in the treatment of erysipelas arising from wounds, I found that the application of it was attended with decided success in destroying the inflammation, and preventing its progress. I was thence induced to try the same remedy in erysipelas of the face arising from constitutional causes. About seven years ago, indeed, I applied it to the extent of half-a-crown on the inflamed part; but, from not watching its effects for several days, I formed an erroneous opinion of its utility.

A few months ago, I attended a young woman, who was under the care of a physician, with erysipelas arising from constitutional causes, nearly covering the whole face like a small mask of the figure of a heart, leaving a border of nearly two inches around it free from inflammation. I wished in this case to try the effects of a partial application of the nitrate of silver: I accordingly applied it one inch upon the inflamed border, and one inch on the adjoining healthy border, on one side of the face, making an escharred surface of four inches in length and two in breadth. This application had the effect of destroying the inflammation and checking its progress on that side; the inflammation spread rapidly on the other side over the whole scalp and neck, and entirely surrounded the escharred surface in every direction; but it did not affect a single point of the healthy skin to which the nitrate of silver had been applied. The disease ran its usual course with severity, the patient having delirium at intervals for five or six days, although every treatment was had recourse to which is usual in similar cases, as general bleeding, blistering the nape of the neck, purgatives, &c.

Having observed in this case that the nitrate of silver had a most decided effect in destroying the inflammation and preventing its progress, and that in five days the eschar was peeling off, leaving the skin white and free from inflammation and swelling, I was led to think that, if it was applied in such cases to the whole inflamed surface and surrounding parts, that it might effectually check the disease in the commencement, and prevent, in a great measure, the severe continued constitutional affection which follows. In this I was not disappointed. In the succeeding cases it will be found that, the moment the local affection is destroyed by the nitrate of silver, the constitutional affection ceases; and that the patient becomes convalescent even in less time than is frequently occupied by the inflammation in running its usual course under any other mode of treatment.

Case I.—Ann Ward, aged forty-seven years, single, and of a delicate habit of body; the catamenia having ceased for three months for the first time; had been poorly for several weeks with a cold and cough, and two or three days ago was seized with a violent shivering, succeeded by fever and pain of the head; soon afterwards the right side of the nose and eyelid became affected with erysipelatous inflammation, for which she took an emetic and purgatives. The inflammation continued increasing for two days, at which period I saw her; it had spread over the whole of the right side of the face and ear, and over more than one-half of the scalp. She complained of violent pain of the head, which was much increased by the severity of the cough; tongue white and loaded; skin hot and dry; pulse 130 in a minute;—she was insensible during the night, and very restless. I took about fourteen ounces of blood from the arm, and directed a dose of calomel, and a purgative with infusion of senna and salts. I desired the head might be shaved, and the head and face washed with soap and water, to remove any oily state of the skin. I then moistened every part of the inflamed surface with water, and applied a long stick of the nitrate of silver in a flat direction on the whole inflamed surface, and a little beyond it on the surrounding healthy skin, leaving no part untouched.

On my visit the following day, I found that the nitrate of silver had had the effect of checking the inflammation on the face so completely that it had not spread to the other side of the nose; but some part of the scalp beyond where the nitrate had been applied had an œdematous feel: although this part was free from redness, I immediately applied the nitrate of silver freely over it, and over the whole scalp, as well as around the left ear, which had not been affected by the inflammation. The patient said the head had a benumbed feel, but was much less painful. There was scarcely any vesication occasioned by the nitrate of silver on the inflamed parts; but on the surrounding skin, where it was applied, there was more. She was delirious in the night, but had been quite collected during the day; the pulse was 104; the tongue rather cleaner; there was less fever, and the patient had been purged freely.

On the second day, there was no increase of the inflammation; the left ear was not in the least affected; there was a little swelling of the eyelid on the left side, arising from the irritation of the nitrate of silver above it. She was better in every respect. There was a little delirium in the night. Pulse 100. The purging medicine was directed to be continued.

The third day after the first application of the nitrate of silver, there was no appearance of inflammation. She had had no delirium in the night; she had perspired freely, and had less fever; the bowels were open. What little vesications there had been were gone, and the eschars were adherent.

On the fourth day, the eschars were separating, leaving the skin underneath free from inflammation. She had no pain from the eschars, but a little stiffness. Pulse ninety-two; tongue rather loaded; no delirium for two nights; no fixed pain of the head; more sleep; cough not quite so troublesome; appetite a little better.

On the sixth day, the pulse was eighty-eight; the skin cool; the bowels open; and the patient was in every respect better.

From this period my patient might be considered convalescent: her cough was rather troublesome for several days; her pulse diminished in frequency, being eighty in a minute on the tenth day. She soon left her room, and required no further attention.

In remarking the above case, it will be perceived that there was no delirium after the external inflammation was subdued by the nitrate of silver, which was decidedly effected on the third day, and that the patient was convalescent in a little more than half the time that the inflammation is in running its usual course; and, consequently, that the disease was cured before that period in which it becomes the most dangerous.

Case II.—Miss Wells, aged thirty, has for several days felt generally unwell, with loss of appetite, debility, and drowsiness. During the last night, she was very restless; and this morning she had considerable heat of skin, with shiverings at intervals: this was succeeded by an erysipelatous inflammation on the nose and left eyelid. The inflammation not being severe, I did not apply the nitrate of silver, expecting it would give way to the antiphlogistic treatment. I took about fourteen ounces of blood from the arm, which caused her to faint. I prescribed an emetic, and some infusion of senna with salts. The inflammation spread very little during the day.

The next morning I learnt that she had had a restless night: her face was very hot and painful, and the pain of the head was violent; there was fever, attended with shivering. The inflammation had spread a little on the right side of the face. The purging medicine was continued. In the evening, the inflammation had spread nearly over the whole of the face; some vesications had formed on the right cheek, and there was an increase of the constitutional symptoms; the pulse was 112. I applied the nitrate of silver, as in the former case, upon and beyond the inflamed surface, except on the eyelids, having found it in former cases to occasion a considerable flow of tears, which, passing over the escharred surface, is rather troublesome, inducing excoriation. The immediate pain given by the application of the nitrate of silver in this instance was severe, and compared by the patient to a scald, and continued for two hours, and in a less degree during the night; the pain of the head, however, gradually abated.

The next morning there was no increase of inflammation; there was still a little headache; pulse ninety-six; a little perspiration; bowels open. In the evening, I applied the nitrate of silver to several parts which appeared a little inflamed.

On the following morning the patient had more headache; the pulse was ninety-two; and, on examination, I found that the inflammation had spread over the scalp, there being a slight degree of redness, and beyond it an œdematous feel. I ordered the head to be shaved, and applied the nitrate of silver on the greatest part of the scalp. In the evening, there was no appearance of inflammation; the patient had experienced very sensible relief from the application of the nitrate of silver; in one hour afterwards the headache quite ceased, and she has since had no return of it.

The next morning my patient was in all respects better; pulse eighty. I applied the nitrate of silver on the remaining part of the scalp, although there was no appearance of inflammation; for as this is more obscure on the scalp in the commencement, and sometimes cannot be ascertained except by the œdematous feel, or by the sensation of pain to the patient when pressed upon by the finger, I would advise the whole scalp to be escharred, if there were any part of it inflamed; the pain from the nitrate of silver is much less here than in other parts, and the immediate benefit in relieving the pain of the head by destroying the inflammatory action is very apparent.

On the fourth morning after the application of the nitrate of silver, the pulse was eighty; the catamenia appeared; the patient felt rather low, and had a little hysterical affection and delirium.

On the fifth morning, the cuticle was separating, and the patient was convalescent. The opening medicine was directed to be continued.

On the eighth day, my patient was sitting up in her room.

On the tenth day, most of the cuticle had separated from the face, leaving it in its natural state.

In the last case, the delirium appeared to be decidedly prevented by the prompt application of the nitrate of silver to the scalp. The remedy also prevented the ears and chin from being in the least affected by the inflammation, and the patient might be considered convalescent on the fifth day. I had much satisfaction in observing the certainty and regularity of the effects of the remedy.

*Nottingham; August,* 1827.

I have great pleasure in adding the following testimony of Dr. Storer, the well-known distinguished physician of this place, to the utility of the nitrate of silver in erysipelas. That testimony will be received with the greatest satisfaction by those who best know Dr. Storer's extreme accuracy in observing facts, and caution in admitting them.

*Nottingham; April 10th,* 1827.

Dear Sir,—Your late Treatise had apprised me of your mode of using lunar caustic in the treatment of certain wounds and ulcers. I had not lost sight of the facts there detailed, and have been gratified to find, by repeated inquiries, that this plan of treatment continues to be confirmed by its success in your hands, and by other surgeons who have adopted it. It is now understood that it is capable of being extended, with signal benefit, to old and extensively ulcerated legs; of which one case that I have seen is certainly an example. In cases of local disease, such success does not exceed the ordinary bounds of expectation; nor is it without analogy to the general application of this substance. When I heard that for some time past you had adopted the same plan of extinguishing cutaneous inflammation, not merely in symptomatic erysipelatous affection, but also in that constitutional erysipelas which is often epidemic, and always ushered in and accompanied with fever, I confess that I could expect no success from a practice militating so directly against the views generally entertained of the nature of this disease, and would give no ear to it without ocular demonstration of its utility.

I have now seen two cases of constitutional erysipelas treated upon this principle; and, as far as two cases well marked will go to authorise a conclusion, mine must be greatly in favour of the practice.

The first was that of a healthy young woman, upwards of twenty years of age, seized with rigor, followed by smart fever, and on the second day by a complete erysipelas of the face, extending to the hair on the forehead. The fever continuing, with a further extension of the inflammation, after bleeding, purgative and saline medicines, and a small vesication having arisen on the cheek, it was judged necessary, on the third day, to apply the caustic over the whole extent of the erysipelas. The patient's own account was, that she suffered considerable pain for ten hours; but from that time the feverish symptoms ceased, and the inflammation was arrested and subdued. When I saw her on the fourth day after the application of the caustic, there was neither fever nor any remains of erysipelas. The face was as black as that of an African; but in a few days more I found her free from all symptoms of disorder, the epidermis peeling off, and the complexion underneath quite natural.

The other case was a fine healthy boy of sixteen months old, who, after a feverish attack, had erysipelas on one hip and thigh, and extending partially to the leg. In this case also the fever and inflammation had subsisted forty-eight hours and upwards before the application of the caustic. The account of the child's mother was, that it cried very much for one hour after, then fell into a long and calm sleep, out of which he awaked without fever, and calling out for food. In three days afterwards, when I saw him, he was quite well, and the scarf-skin of the inflamed parts separating.

This is the extent of what I have observed of this novel mode of treating erysipelas, and which has surprised as it has satisfied me. Sincerely wishing success to your endeavours to improve the practical part of your profession,

I am, dear sir, yours faithfully,

JOHN STORER.

---

**[In several of our recent Numbers, we have given papers from the American Journals. Some of these periodicals contain numerous original communications of interest, and it is our intention hereafter to insert such of them as are of sufficient importance and as our limits will admit of, immediately after the papers of our own contributors.]**

